# Supplementary figures and images for: Transcriptome Analyses Identify an RNA Binding Protein Related Prognostic Model for Clear Cell Renal Cell Carcinoma
Source: Front Genet. 2021 Jan 7;11:617872. doi: 10.3389/fgene.2020.617872 (PMC7817999; doi:10.3389/fgene.2020.617872)

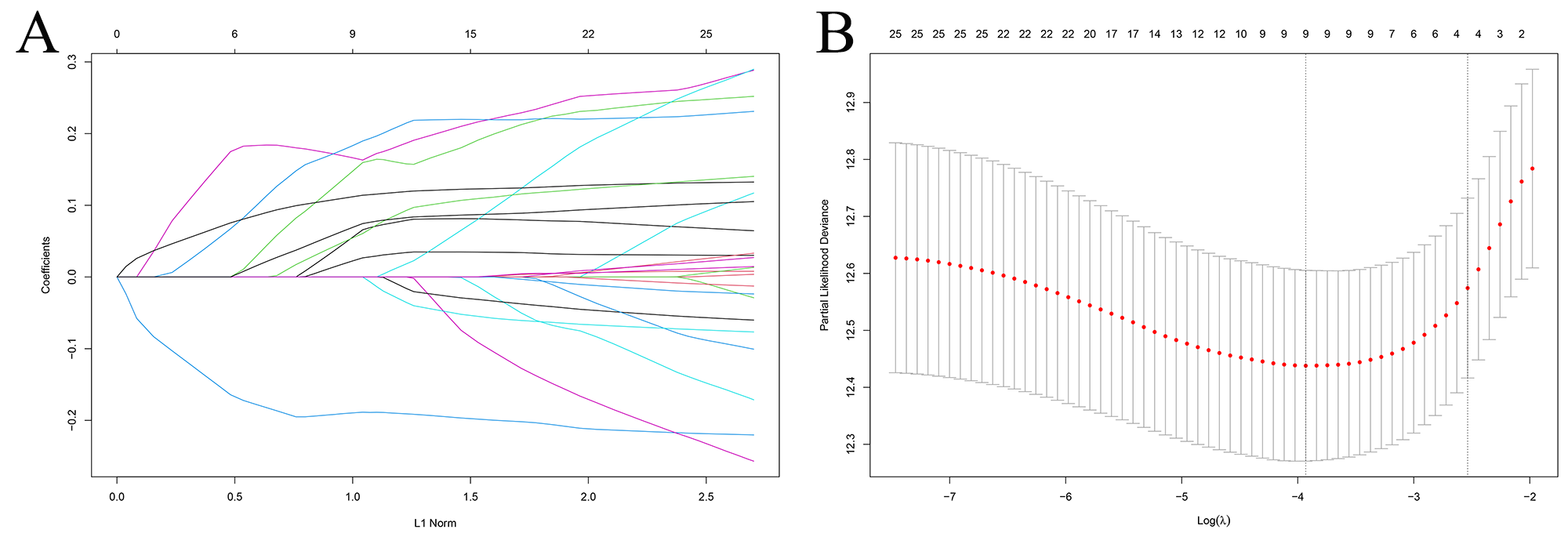

Supplement: Supplementary file 1 [file Image_1.TIF]
