# Supplementary material for: Transcriptome Analyses Identify an RNA Binding Protein Related Prognostic Model for Clear Cell Renal Cell Carcinoma
Source: Front Genet. 2021 Jan 7;11:617872. doi: 10.3389/fgene.2020.617872 (PMC7817999; doi:10.3389/fgene.2020.617872)
Supplement: Supplementary file 2 [file Table_1.DOCX]

| **Variables** | | **TCGA_KIRC** | **E-MTAB-1980** |
| --- | --- | --- | --- |
| **Age** | >65 | 186 | 44 |
|  | ≤65 | 353 | 57 |
| **Gender** | Male | 353 | 77 |
|  | Female | 186 | 24 |
| **Grade** | Grade 1&2 | 249 | 72 |
|  | Grade 3&4 | 282 | 27 |
| **Stage** | Stage Ⅰ&Ⅱ | 331 | NA |
|  | Stage Ⅲ&Ⅳ | 1581 | NA |
| **T stage** | T 1&2 | 349 | 79 |
|  | T 3&4 | 190 | 22 |
| **N stage** | N 0 | 241 | 94 |
|  | N 1&X | 298 | 7 |
| **M stage** | M 0 | 428 | 89 |
|  | M 1&X | 109 | 12 |

Table S1. Clinical parameters of patients.
